# Effect of nipple shield use on milk removal: a mechanistic study

**DOI:** 10.1186/s12884-020-03191-5

**Published:** 2020-09-07

**Authors:** Viviane Silva Coentro, Sharon Lisa Perrella, Ching Tat Lai, Alethea Rea, Kevin Murray, Donna Tracy Geddes

**Affiliations:** 1 School of Molecular Sciences, Faculty of Science, The University of Western Australia, M310, 35 Stirling Highway, Western Australia 6009 Crawley, Australia; 2 Mathematics and Statistics, School of Engineering and Information Technology, Murdoch University, 90 South Street, Western Australia 6150 Murdoch, Australia; 3 School of Population and Global Health, Faculty of Health and Medical Sciences, Population and Global Health, The University of Western Australia, M431, 35 Stirling Highway, Western Australia 6009 Crawley, Australia

**Keywords:** Nipple shield, Nipple pain, Pumping, Milk removal

## Abstract

**Background:**

Concerns about reduced milk transfer with nipple shield (NS) use are based on evidence from studies with methodological flaws. Milk removal during breastfeeding can be impacted by infant and maternal factors other than NS use. The aim of this study was to control electric breast pump vacuum strength, pattern and duration across multiple study sessions to determine if NS use reduces milk removal from the breast.

**Methods:**

A within-subject study with two groups of breastfeeding mothers (infants < 6 months) were recruited; Control Group (CG): no breastfeeding difficulties; Pain Group (PG) used NS for persistent nipple pain. Mothers completed three randomised 15 min pumping sessions using the Symphony vacuum curve (Medela AG); no NS, fitted NS, and a small NS. Sessions were considered valid where the applied vacuum was within 20 mmHg of the set vacuum. Milk removal was considered as pumped milk volume, and also percentage of available milk removed (PAMR), which is calculated as the pumped volume divided by the estimated milk volume stored in the breast immediately prior to pumping.

**Results:**

Of 62 sessions (all: *n* = 31 paired sessions) a total of 11 paired sessions from both PG (*n* = 03) and CG (*n* = 08) were valid (subset) with and without a fitted NS. Only 2 small shield sessions were valid and so all small shield measurements were excluded. Both pumped volumes and PAMR were significantly lower with NS use for all data but not for subset data. (All: Volume and PAMR median: no NS: 76.5 mL, 69%, Fitted NS: 32.1 mL, 41% respectively (volume *p =* 0.002, PAMR *p =* 0.002); Subset: Volume and PAMR median: no NS: 83.8 mL, 72%; Fitted NS: 35.2 mL, 40% (volume *p =* 0.111 and PAMR *p =* 0.045). The difference in PAMR, but not volume, was statistically significant when analysed by linear mixed modelling. A decrease of 10 mmHg was associated with a 4.4% increase in PAMR (*p* = 0.017).

**Conclusions:**

This experimental data suggests that nipple shield use may reduce milk removal. Close clinical monitoring of breastfeeding mothers using nipple shields is warranted.

## Background

It is well known that early cessation of breastfeeding impacts both long and short-term health outcomes for the infant and mother [1, 2]. Nipple pain is one of the most common causes of mothers stopping breastfeeding earlier than planned [3–5]. The causes of nipple pain are varied and may be multifactorial, including suboptimal positioning and attachment, bacterial infection and vasospasm [6–8]. When nipple pain is unable to be resolved by conventional methods a nipple shield may be used [9]. A nipple shield is a thin flexible silicone aid that is placed over the nipple and areola prior to breastfeeding, providing a physical barrier with the aim of increasing maternal comfort while enabling continued breastfeeding. Many mothers continue using nipple shields as they are perceived to be helpful [10, 11]. However, professional opinion remains divided on the use of nipple shields due to concerns about reduced milk transfer to the infant, altered infant sucking [12], and shorter breastfeeding duration [13, 14], although the latter is confounded by the fact that nipple shields are typically used by mothers experiencing breastfeeding problems [15].

Concerns about reduced milk transfer are based on studies performed more than three decades ago where both breastfeeding and breast pump use with a nipple shield resulted in lower milk transfer volumes than when breastfeeding or pumping without a nipple shield [12, 16]. Methodological issues such as small sample size, sampling prior to the establishment of a full milk supply, and absence of confirmation of adequate milk supply all call into question the validity of the reported results [10]. Further, in the pumping study, it was assumed that the breast was completely drained at the end of the pumping session, the duration of pumping prior to milk ejection was not accounted for, and achievement and maintenance of a set cycling pressure and pattern were not confirmed [16].

Assessment of milk removal with and without nipple shield use on can be confounded by variable infant sucking characteristics between study sessions, such as suck burst, pause and total feed durations, intraoral vacuum levels and infant alert state, satiety and age [17]. Also, it is not known if milk transfer volumes differ between women with and without nipple pain. We conducted a mechanistic study to exclude confounding infant factors, controlling and replicating breast pump vacuum strength, pattern and duration across study sessions to determine the effect of nipple shield use on milk removal during pumping in mothers with and without nipple pain.

## Methods

### Participants

Breastfeeding mothers of term healthy infants of ages 1 to 6 months were recruited through the Australian Breastfeeding Association, and international board certified lactation consultants based at the Breastfeeding Centre of Western Australia, King Edward Memorial Hospital or in the community between August 2016 and July 2018. Mothers were predominantly breastfeeding (i.e. feeding ≤ 1 bottle of infant formula per day) and had previous experience of using a breast pump. We recruited two groups: a Pain Group (PG) where mothers were using a nipple shield to manage unexplained nipple pain, and a Control Group (CG) where the mothers had no breastfeeding difficulties.

PG inclusion criteria were: mothers with persistent nipple pain during breastfeeding despite professional lactation advice. CG inclusion criteria: mothers with no breastfeeding pain or difficulties. PG and CG exclusion criteria were: mothers with a diagnosed cause of nipple pain such as infection or nipple vasospasm, previous breast and/or nipple surgery and/or piercings, mothers < 18 years of age, mothers unable to read and speak English without assistance, birth < 37 completed weeks gestation, mothers of infants with an oral anomaly, prior oral surgery and/or a diagnosed health condition. Mothers completed a demographic questionnaire at recruitment.

The study was granted approval by The University of Western Australia (RA/4/1/7863). Mothers provided written informed consent prior to participation.

## Study design

A within-subject study was conducted in a laboratory at The University of Western Australia with mothers required to attend three study sessions. At each session one breast was pumped under one of three randomly assigned conditions: (1) with a fitted nipple shield i.e. nipple shield diameter ≥ 4 mm nipple diameter, (2) without a nipple shield, and (3) with a small nipple shield (nipple shield diameter < 4 mm nipple diameter, or 16 mm diameter nipple shield if nipple diameter ≤ 12 mm). The small nipple shield was used to determine whether nipple shield sizing may explain differences in milk removal with nipple shield use. The same breast was pumped at each visit; the CG mothers’ study breast was randomised, and PG mothers pumped the most painful breast as the indication for nipple shield use was nipple pain.

The pump used was a custom-made software-controlled breast pump (Lactasearch, Medela AG, Baar, Switzerland) that applied the Symphony vacuum curve, with a vacuum range of 0 to -250 mmHg and frequency range of 120 cycles/min for the stimulation phase and 48 to 72 cycles/min for the expression phase. The applied vacuum was measured throughout the pumping sessions and data recorded using the software package DIAdem (version 11.1, National Instruments, Texas, USA, 2009). Mothers used a breast shield that was connected to a collection tube with expressed milk delivered to a bottle placed on a custom built continuous weighing device (ShowMilk, Medela AG, Switzerland; resolution 0.1 g, accuracy ± 0.02% to a maximum 2 kg).

Nipple diameters were measured using electronic callipers (CE Carbon Fiber Composites Digital Calliper, accuracy ± 0.2 mm, Anhui, China) before pumping to determine the appropriate nipple shield size. Pre and post pumping milk samples (< 1.0 mL) were collected in 5 mL plastic tubes (Techno Plas, St Marys SA, Australia). The cream content of the milk was measured using the Crematocrit method [18], and the degree of breast fullness was calculated using Crematocrit and 24 h milk profile results [19].

Central placement of the nipple shield and breast shield over the nipple were confirmed before commencing pumping with a stimulation pattern, with the vacuum setting immediately adjusted to the mother’s maximum comfortable vacuum level. The expression pattern commenced when milk flow was visually detected, or at 2 min if milk flow had not occurred. The vacuum setting was again adjusted to the mother’s highest comfortable vacuum level and applied for 15 min. For each mother, the maximum comfortable vacuum level selected at the first pumping study session was set for subsequent sessions.

Mothers rated the severity of nipple pain experienced during each pumping session using the Visual Analogue Scale (VAS) [20, 21] and McGill Pain Questionnaire (MPQ) [22].

## Measurement of milk removal from the breast

Maternal 24 h milk profile measurements were completed in mothers’ homes within 14 days of the study sessions using electronic scales sensitive to 2 g (Medela BabyWeigh Scales, Medela AG, Baar, Switzerland). Milk production was determined by weighing the infant [23] pre and post breastfeeds and by weighing milk collection bottles pre and post any pumping sessions over a 24 h period. Milk samples (< 1.0 mL) were collected before and after every breastfeed and pumping session and frozen for later analysis. All breastfeeding and pumping measurements were expressed in grams and considered equivalent to mL (1.03 g mL^− 1^ = 1.0 mL of breast milk) [24].

As breast storage capacities differ between women and the volume of milk available in the breast varies over time, the volume of milk removed by breastfeeding or pumping is not an accurate indicator of the degree to which the breast was emptied [18, 25]. Therefore, each mother’s breast storage capacity, degree of breast fullness, and percentage of available milk removed (PAMR) were estimated as described by Kent et al. [26], thereby allowing more accurate comparisons between pumping sessions. For example, one mother may have a breast storage capacity of 100 mL, and another mother a breast storage capacity of 200 mL. If these two mothers pump from a full breast and each removes 50 mL, the first mother will have removed 50/100 mL or 50% (50% PAMR) and the second mother 50/200 mL or 25% of the available milk (25% PAMR). The PAMR reflects the degree of emptying and therefore effectiveness of milk removal more accurately than volume alone.

## Sample size determination

The primary endpoint of this study is total milk volume removed during pumping with and without a nipple shield. Sample size determination for this project was completed using the data source of McClellan et al. [5] where raw data was sourced from 21 women reporting nipple pain and compared with 21 mothers without nipple pain with regard to 24 h milk production, milk transfer volumes, and estimated milk available in the breast (mL). The sample size was calculated using a bootstrap approach where it considered two feeds and then added a nipple shield effect in one of the feeds. For the purpose of this sample size determination, it was assumed that nipple shield use decreases milk removal from the breast. All analyses were performed using R, and a sample size of 30 (*n* = 30) was recommended to detect an average significant difference of 20 ± 5 mL (power: 0.83, alpha: 0.05) between sessions with and without nipple shield use.

## Statistical methods

An interim review of the data showed that for 20 of 31 pumping sessions where a nipple shield was used, the applied vacuum did not reach the set vacuum. For these 20 sessions, we observed movement of the nipple shield and the applied vacuum was often more than 20 mmHg weaker than the set vacuum. Valid data was difficult to obtain because the differences in vacuum could not be resolved technically. Therefore, analysis was carried out on all data and on the valid data (subset analysis). This subset was defined as mothers who had complete data for paired sessions (with and without fitted nipple shield use) where the applied maximum comfortable vacuum was within 20 mmHg of the set vacuum across the sessions.

The data was analysed using linear mixed models with either PAMR or milk volume as the response variable and group (control or pain), nipple shield (none or fitted nipple shield), degree of breast fullness (milk volume models only) and applied vacuum (all models) as explanatory variables with a random effect for mother. Only two mothers had valid data for small nipple shield, fitted nipple shield and no nipple shield pumping sessions. Therefore data for the small nipple shield sessions were excluded as the data were insufficient to determine whether a use of a small nipple shield impacted milk removal. Model selection was carried out on all models in order to retain only significant variables in the final models.

Control and Pain groups 24 h milk productions, VAS and MPQ scores were compared using a Wilcoxin signed rank test. Control and Pain groups were compared using a Wilcoxin signed rank test for different conditions (with and without the nipple shield). Categorical data for demographic characteristics were compared using Fisher’s exact test. Paired t-tests were used to compare of pumping characteristics with and without nipple shield use.

Descriptive statistics are presented as medians (IQR) for numerical variables and frequencies and percentages for categorical variables. Model estimates are presented as estimate (standard error). The significance level was set at 0.05 and the analysis was carried out in R version 3.5.1.

## Results

Maternal characteristics are shown in Table 1. Data were collected for up to three pumping sessions from 31 mothers in total (PG *n* = 09, CG *n* = 22), and results reported for 31 paired sessions of pumping without and with a fitted nipple shield. Valid data were obtained for 11 paired sessions to provide subset data for 11 mothers (PG *n* = 03, CG *n* = 08).

**Table 1:** Maternal and pumping characteristics for Pain and Control groups

**Maternal characteristics**	‘all data’				‘subset data’	
	**PG (*n* = 09)**	**CG (*n* = 22)**	***p*-value**		**PG (***n*** = 03)** median only	**CG (***n*** = 08)**
Maternal age; years	30 (5)	33 (3)	0.03		25.7	33.3 (91.6)
Primipara; n (%)	7 (78)	14 (70)	0.67		3 (100)	6 (86)
Birth gestation; weeks	39 (1)	40 (2)	0.39		39	39 (3)
Birth mode vaginal; n (%)	5 (56)	14 (64)	0.70		2 (67)	4 (57)
Postpartum time; weeks	11.0 (4.4)	15.1 (11.1)	0.04		11.0	15.7 (11.3)
24 h milk production; mL	768 (178)	739 (305)	0.89		654	622 (98)
Postpartum time of milk production; week	6.7 (2.4)	13.4 (6.6)	0.04		6.6	12.6 (6)
**Pumping characteristics**						
MCV, no nipple shield; mmHg	-262 (29)	-232 (59)	0.14		-240	-197 (72)
MCV, with nipple shield; mmHg	-260 (26)	-236 (59)	0.20		-238	-196 (76)
PAMR, no nipple shield; %	55 (24)	73 (29)	0.31		50	76 (18)
PAMR, with nipple shield; %	44 (32)	41 (47)	0.91		37	40 (71)
Volume, no nipple shield; mL	59 (60)	82 (60)	0.56		57	102 (52)
Volume, with nipple shield; mL	37 (46)	31 (62)	0.67		37	33 (104)

Results are reported as median (IQR) for maternal age, birth gestational age, 24 h milk production (range), time of measuring milk production, maximum comfortable vacuum (MCV), percentage of available milk removed (PAMR) and volume with and without nipple shield (NS). Parity and birth mode are reported as number and percentage (%)

The 24 h milk production median volumes were similar between groups (Table 1). For the subset data median 24 h milk production were 654 mL for PG and 622 mL for CG.

For pumping sessions with a nipple shield, both volume and PAMR were lower for both all and the subset data (Table 2).

**Table 2:** Degree of fullness, applied vacuums, milk volume and PAMR with and without nipple shield

	No Nipple Shield	Nipple Shield	*p*-value
Degree of fullness			
All data	0.76 (0.42)	0.73 (0.50)	0.863
Subset	0.81 (0.25)	0.73 (0.23)	0.375
Applied vacuum (mmHg)			
All data	-235 (60)^a^	-186 (73)	0.019
Subset	-218 (66)^a^	-220 (59)	0.770
Volume (mL)			
All data	76.5 (63.1)	32.1 (58.4)	0.002
Subset	83.8 (56.7)	35.2 (81.6)	0.111
PAMR (%)			
All data	69 (33)^a^	40.7 (40)^a^	0.002
Subset	72 (19)^a^	40.0 (49)^a^	0.045

^**a**^One record with missing data

Pre-pumping degree of fullness, applied pumping vacuums, milk volume and percentage of available milk removed (PAMR) with and without nipple shield presented reported as raw data as median (IQR) for all data (*n* = 31) and subset data (*n* = 11)

**Fig. 1:**
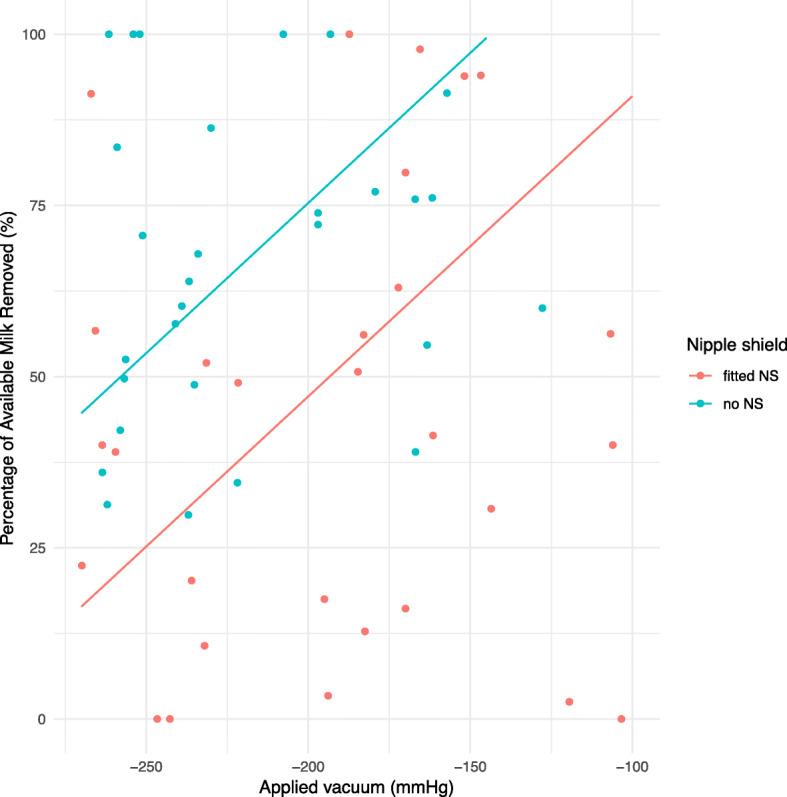
Percentage of available milk removed (%) and applied vacuum (mmHg) with and without fitted NS

When considering all data, modelling showed that PAMR was lower with a nipple shield. When a fitted nipple shield was used the PAMR was 24.0 (6.8) percentage points lower than if no nipple shield was used (*p =* 0.002). For the subset data when a fitted nipple shield was used the PAMR was 28.2 (11.6) percentage points lower than if no nipple shield was used (*p =* 0.045). A decrease of 10 mmHg in applied vacuum was associated with a 4.4 (1.4) percentage point increase in PAMR (*p =* 0.017). When considering the subset data only, PAMR was associated with nipple shield use and applied vacuum (Fig. 1).

When considering all data, milk volume was associated with nipple shield use and degree of fullness of the breast. If a fitted nipple shield was used then the milk volume was 36.0 mL (10.3) lower than if no nipple shield was used (*p =* 0.002). A 0.1 unit increase in degree of fullness was associated with a 9.3 mL (2.7) increase in milk removed (*p =* 0.002). For the subset data, nipple shield use was not associated with milk volume (*p =* 0.11), and a 0.1 unit increase in degree of fullness was associated with a 15.1 mL (5.3) increase in milk removed (*p =* 0.02).

Mothers in the PG scored higher and more variable pain scores than mothers in the CG during pumping, although more than half of PG scores indicated low pain levels (Table 3) [27].

**Table 3:** VAS and McGill median (IQR) scores for Pain and Control groups

	Pain group	Control group	*p*-value
**McGill no NS**	7 (12)	0 (4)	0.001
**McGill NS**	6 (10)	0 (4)	0.004
**VAS no NS**	3 (3.5)	0 (0.5)	0.01
**VAS NS**	1.5 (1.3)	0 (0.7)	0.01

## Discussion

Findings from this study suggest that use of a fitted nipple shield during pumping reduces effectiveness of milk removal. Also, both an increased degree of breast fullness, and increased strength of applied pumping vacuum are associated with a greater pumped milk volume.

The volume of milk removed from the breast during pumping is not an accurate indicator of pumping effectiveness as it does not account for the volume of milk in the breast prior to milk removal [19]. However, consistent with the percentage of available milk removed from the breast, the volume was also reduced, although this was not statistically significant when accounting for degree of breast fullness and applied vacuum. This is likely because the sample size of the subset group did not provide sufficient power to detect a statistically significant difference. Auerbach [16] reported reduced milk volume when pumping with a nipple shield, however her study assumed a constant application of vacuum with a nipple shield. The subjects included 25 mothers ranging from 6 weeks to 14 months of lactation. While differences in pumped milk volumes were solely attributed to nipple shield use, the wide span of lactation stages may have contributed several confounders such as reduced milk supply associated with complementary feeding of solids and/or formula or weaning [28]. Woolridge [12] demonstrated reduced milk volume transfer during breastfeeding with a rubber Mexican Hat nipple shield, but no significant difference when a thin latex nipple shield was used. The decreased milk transfer associated with Mexican Hat nipple shield use was attributed to observed altered sucking patterns including a faster suck rate and longer pauses, and possible failure of the milk ejection reflex. Neither study accounted for the starting degree of fullness of the breast. Our findings have shown that the degree of fullness influences the volume pumped regardless of nipple shield use.

Pumping with an appropriately sized nipple shield reduced the effectiveness of milk removal with approximately 25% less of the available milk removed than when pumping without a nipple shield (Table 2). The analysis included mothers that experienced nipple pain when breastfeeding and during the pumping session. While there were no differences in the effectiveness of milk removal between the mothers with pain and those without, ratings of pain were significantly higher than the control group (Table 3). The pain scores for the women experiencing pain were relatively low so it is unlikely that maternal nipple pain inhibited the milk ejection reflex and therefore milk removal [29, 30].

Greater proportions of the available milk were removed from the breast when applied pumping vacuum levels were stronger, regardless of whether a nipple shield was used (Fig. 1). This finding is consistent with previous pumping studies that have had the capacity to measure applied vacuum [31], contributing further evidence for the importance of applying the highest comfortable vacuum to enhance pumping effectiveness.

The results of this study also indicate that the degree of fullness or amount of milk available in the breast prior to pumping is related to the volume of milk removed such that the fuller the breast, the larger the volume of milk removed. Our findings concur with previous studies that have demonstrated an association between degree of breast fullness and pumped milk volume [32, 33]. These findings highlight the importance of accounting for maternal factors such as 24 h milk production and degree of breast fullness when evaluating the effectiveness of milk removal from the breast, whether it be through pumping or breastfeeding.

### Study limitations

The findings of this study must be viewed in light of the small sample of valid data, that was due to technical difficulties. While an association between nipple shield use and milk removal during pumping was observed in the subset of 11 participants with valid data, analysis based on the estimated required sample size of 30 would increase confidence in these results.

While this study was not designed to explore maternal nipple pain and its impact on milk removal during pumping, we recognise that pain is complex and may be impacted by perinatal mood disorders, previous and current pain experiences, and factors such as breast pump vacuum settings. Detailed studies of pain in breastfeeding mothers are needed to better understand its aetiology and impact on milk removal.

By the very nature of a mechanistic study, our results may inform our understanding of milk removal from the breast with a nipple shield in situ, but cannot be directly applied to the breastfeeding dyad. A clinical study of infant sucking dynamics during breastfeeding with and without a nipple shield is required to determine the impact of nipple shield use on milk removal.

## Conclusions

Evidence from this mechanistic pumping study suggests that nipple shield use reduces milk removal from the breast. A detailed study of nipple shield use in breastfeeding dyads is required to determine the impact of nipple shields on infant sucking and milk removal from the breast. Breastfeeding mothers using a nipple shield should be monitored for adequate breast emptying and milk production.

## Data Availability

All datasets generated or analysed during this study are available from the corresponding author on request.

## Abbreviations

PG: Pain Group
CG: Control Group
NS: Nipple shield
VAS: Visual Analogue Scale
MPQ: McGill Pain Questionnaire
BPI: Brief Pain Questionnaire
PAMR: Percentage of available milk removed
MCV: Maximum comfortable vacuum
SD: Standard deviation
